# TiO_2_ Nanotopography-Driven Osteoblast Adhesion through Coulomb’s Force Evolution

**DOI:** 10.1021/acsami.2c07652

**Published:** 2022-07-22

**Authors:** Jiajun Luo, Shudong Zhao, Xiangsheng Gao, Swastina Nath Varma, Wei Xu, Maryam Tamaddon, Richard Thorogate, Haoran Yu, Xin Lu, Manuel Salmeron-Sanchez, Chaozong Liu

**Affiliations:** † Division of Surgery & Interventional Science, Royal National Orthopaedic Hospital, University College London, Stanmore HA7 4LP, U.K.; ‡ Key Laboratory for Biomechanics and Mechanobiology of Ministry of Education, Beijing Advanced Innovation Center for Biomedical Engineering, School of Biological Science and Medical Engineering, Beihang University, Beijing 100083, China; § Beijing Key Laboratory of Advanced Manufacturing Technology, Faculty of Materials and Manufacturing, Beijing University of Technology, Beijing 100124, China; ∥ Beijing Advanced Innovation Center for Materials Genome Engineering, Institute for Advanced Materials and Technology, State Key Laboratory for Advanced Metals and Materials, University of Science and Technology Beijing, Beijing 100083, China; ⊥ London Centre for Nanotechnology, University College London, London WC1H 0AH, U.K.; # Institute of Bioengineering, College of Chemical and Biological Engineering, Hangzhou Global Scientific and Technological Innovation Center, Zhejiang University, Hangzhou 310027, China; ¶ Centre for the Cellular Microenvironment, University of Glasgow, Glasgow G12 8LT, U.K.

**Keywords:** Ti implant, nanotopography, cell−material interaction, protein adsorption, cell adhesion

## Abstract

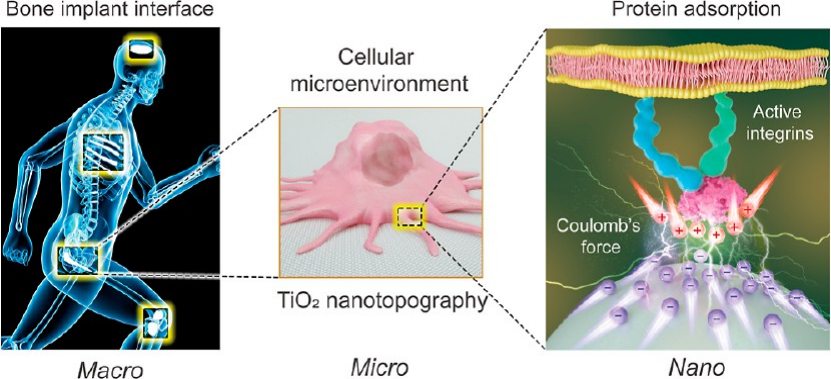

Nanotopography is an effective method to regulate cells’ behaviors to improve Ti orthopaedic implants’ in vivo performance. However, the mechanism underlying cellular matrix–nanotopography interactions that allows the modulation of cell adhesion has remained elusive. In this study, we have developed novel nanotopographic features on Ti substrates and studied human osteoblast (HOb) adhesion on nanotopographies to reveal the interactive mechanism regulating cell adhesion and spreading. Through nanoflat, nanoconvex, and nanoconcave TiO_2_ nanotopographies, the evolution of Coulomb’s force between the extracellular matrix and nanotopographies has been estimated and comparatively analyzed, along with the assessment of cellular responses of HOb. We show that HObs exhibited greater adhesion and spreading on nanoconvex surfaces where they formed super matured focal adhesions and an ordered actin cytoskeleton. It also demonstrated that Coulomb’s force on nanoconvex features exhibits a more intense and concentrated evolution than that of nanoconcave features, which may result in a high dense distribution of fibronectin. Thus, this work is meaningful for novel Ti-based orthopaedic implants’ surface designs for enhancing their in vivo performance.

## 1 Introduction

Titanium-based biomaterials have been widely applied in orthopaedic implants due to the several nanometers thick TiO_2_ film providing a successful biocompatibility.^1^ However, this natural formed film is biologically inert and leads to poor bone conductive and cell adhesion.^2^ Cell–material interaction is fundamental but vital to Ti-based orthopaedic biomaterials’ success.^3−5^ The interaction represents the attachment of adherent cells to a material surface and their related cell behaviors such as proliferation, differentiation, migration, or apoptosis.^6−10^ Cell responses to their surrounding microenvironment are mediated by the extracellular matrix (ECM), providing a natural web of intricate nanofibers to support cells and present a cell-instructive microenvironment to guide cell behaviors.^11,12^ Among the ECM proteins, fibronectin (FN) is a large dimeric glycoprotein, initially adsorbed on the material surface,^13^ which is able to ligate to cell receptors termed integrins through a cell-binding domain, FNIII9–10, containing the arginine–glycine–aspartic (RGD) sequence. Thus, FN plays a key initial mediator role with several conformation, and it has been proven that the adsorption of FN on a biomaterial is an effective approach to improve its biofunctional performance.^13−16^

Materials science offers the possibility to precisely engineer the ECM–material interface through the control of chemistry,^7,17^ stiffness,^18,19^ and topography down to the nanoscale^20,21^ to instruct cell behaviors though anchored proteins. Nanotopographical materials are particularly interesting as they are in the same size range as protein molecules of interest.^22,23^ In terms of nanotopographical fabrication, electron beam lithography and block copolymer technique anodization methods have been used to generate nanopits^21,24^ and nanopillars^25−27^ to investigate focal adhesion (FA),^22^ filopodia sense/extension,^28^ and osteoinduction.^29^ It is remarkable that to make sense to orthopaedics, it is important to fabricate into appropriate materials, such as Ti;^30^ this presents challenges in terms of high-fidelity nanoscale fabrication.

To understand the nanotopographical mechanisms of cell control on Ti, theoretical calculations between the ECM and Ti nanorough topographies have been conducted. Electrostatic interaction is the dominant factor, and it was concluded that the binding of a peptide to a Ti-based substrate is strengthened by the ionic interactions of charged atoms and polar interactions of neutral atoms.^31,32^ In specific, the interaction between the negatively charged Ti surface and a negatively charged plasma membrane is mediated by charged proteins with a distinctive quadrupolar internal charge distribution.^33^ Regarding the nanofeatured Ti, nanorough topography creates a surface with an enhanced electric field strength that is strongly attractive (electrostatic attraction, Coulomb’s force) to proteins due to the increased curvature of edge.^23^ Furthermore, the higher the surface charge density, the more the cation will act as an attractant mediator between FN and Ti.^33−35^ This charge-dominated mechanism is proposed through dynamics simulation in nanotopography–FN interactions in the nanoscale. However, it is unfeasible to investigate the FN adsorbing in situ because of the interference of charge caused by introducing a test probe into the FN–substrate system. Thus, the combination of experimental investigation and theoretical calculation is a critical approach for further revealing the mechanism of ECM–nanotopography interactions.

According to this theory, the electric field intensity is determined by the curvature of the nanotopographical features. Thus, this is an essential parameter to consider to achieve optimal, consistent electrostatic attraction—Coulomb’s forces to enhance bone cell interaction with Ti surfaces. Hence, we fabricated nanoflat, nanoconvex, and nanoconcave topographies in TiO_2_ as such shapes have been implicated in primary human osteoblast (HOb) initial attachment. A model of FN adsorption was then determined to describe the variation of electric field and the evolution of Coulomb’s electrostatic attraction on the nanotopographies before the initial adhesion behavior of HObs was analyzed. The cytoskeleton of cells on nanoconvex topographies demonstrated well-organized arrangement along with the formation of super mature FA formation (super mature adhesions have a length >5 μm). Cells on nanoconcave topographies had a decreased F-actin organization and smaller FA, typically 2–3 μm in length. Poor adherent behavior of cells on nanoflat substrates was observed. Cellular filopodia and morphological features were observed by scanning electron microscopy (SEM), shown identical spreading and adhesion behavior with confocal images. Our modeling combined to these results supported the hypothesis that Coulomb’s force produced by nanoconvex is not only more intense but also constantly attracting FN and leads to an increased FN ligand density at the tip region on each convex subunit. This high dense adsorbed FN leads to a higher density of ligand (integrin), thus triggering mature FA formation and further improved adhesion of cells.

## 2 Experimental Section

### 2.1 Nanotopography Fabrication and Characterization

TiO_2_ nanoconvex and nanoconcave used in this work were fabricated on pure titanium foil (99.6+% purity, 1.5 mm thickness, GoodFellow) using anodization. In brief, the titanium foil was immersed in ethylene glycol (Fisher Chemical) with 0.5 wt % NH_4_F (>98.0%, Fisher Chemical) as anode, the countering cathode was a graphite sheet. Under a constant 40 V (DC), the titanium foil was immersed into distilled water and vibrated in an ultrasonic cleaner to polish titanium. Again, the polished titanium foil was anodized in similar conditions to generate a TiO_2_ tubular film. After that, surfaces coated with epoxy glue (Araldite) on titanium disks were applied to peel the TiO_2_ tubular film off the titanium substrate. Nanoflat was fabricated on silicon wafer by an electron beam evaporator, with a layer of Ti (∼50 nm, thickness ± 10%) deposited by the electron beam evaporation method with 0.02 nm/s deposition rate. After evaporation, the nanoflat titanium silicon wafer was cut into 10 × 10 mm^2^ squares.

### 2.2 Scanning Electron Microscopy

Nanotopograph arrangements were subsequently measured by SEM (FEI Nova, under 10 kV, WD = 10 mm) after sputter-coated with 3 nm Au. For cell SEM imaging, HOb cells were seeded on the nanotopographies at 2000 cells/cm^2^ and were fixed in 1.5% glutaraldehyde/0.1 M sodium cacodylate buffer for 1 h at 4 °C. After fixation, cells were then washed three times in 0.1 M sodium cacodylate buffer before incubation in 1% osmium tetroxide/0.1 M sodium cacodylate buffer. Afterward, nanotopographies were washed three times with deionized (DI) water and stained with 0.5% uranyl acetate/distilled water for 1 h in the dark, and followed by the dehydration procedure through an ethanol gradient (30, 50, 70, 90, and 100% ethanol). Samples were loaded onto a critical point dryer (liquid CO_2_) for 1 h 30 min and then given a gold/palladium coating using a POLARON SC515 SEM COATER. High-resolution secondary electron of cell nanotopography interactions imaging was performed at the Imaging, Spectroscopy and Analysis Centre (ISAAC) at the University of Glasgow, UK. The images were acquired with a Zeiss Sigma VP Field Emission scanning electron microscope under high vacuum conditions using 5 kV accelerating voltage, an aperture size of 30 mm, and at a working distance of 5 mm.

### 2.3 X-ray Photoelectron Spectroscopy

The chemical composition of nanotopographies was measured by X-ray photoelectron spectroscopy (XPS), and XPS was carried out with a two chamber Thermo K-alpha spectrometer using a monochromated Al Kα X-ray source (1486.6 eV) in the constant analyzer energy mode. X-rays were focused to a 400 μm spot at the nanotopography surface, which defined the analysis area. Sample charging was prevented by use of a dual beam flood gun. High-resolution core line spectra, were recorded at 20 eV pass energy, and survey spectra were recorded at 150 eV pass energy.

### 2.4 Atomic Force Microscopy

The precise topographical features of nanoconvex and nanoconcave were examined by atomic force microscopy (AFM, Bruker AXS Dimension Icon) with a ScanAsyst cantilever (0.4 N/m) in the PeakForce Tapping mode. High resolution was carried out for nanoflat topography.

### 2.5 Kelvin Probe Force Microscopy

Surface potential was characterized by Kelvin probe force microscopy (KPFM, Bruker AXS Dimension Icon) with sample bias model (*W*_sample_ = *W*_tip_ + potential). For nanoconvex and nanoconcave, the Kelvin probe by used a PFQNE-AL cantilever 0.8 N/m in PeakForce Tapping Kelvin probe AM. The tip function was 4.4 eV. KPFM surface charge for nanoflat was applied with the blunter tip that gives lower resistance, and with samples bias, the tip work function was 4.29 eV calibrated on freshly cleaved highly oriented pyrolytic graphite (HOPG) (4.6 V). In situ FN distribution on nanoflat, nanoconvex, and nanoconcave were characterized by AFM. FN from human plasma was adsorbed on substrates by immersing the substrates in FN solution at a concentration of 5 μg/mL in phosphate-buffered saline (PBS). To comparable analysis, a control group was performed to test nanoflat, nanoconvex, and nanoconcave topographies in pure PBS solution.

### 2.6 HOb Seeding

Nanoflat, nanoconvex, and nanoconcave topographies were cleaned using distilled water and sterilized with EtOH. After drying in a hood, the nanotopographies were adsorbed with human plasma FN (from Sigma Merck) solution at 5 μg/mL in PBS buffer for 20 min. The solution was then adsorbed onto the surface, and the specimens were dried in a hood. HObs (from Sigma Merck) were cultured on specimens for 3 h with 120 μL/sample density. Each specimen was seeded with 1200 cells were maintained with Dulbecco’s modified Eagle medium contains with 1% penicillin without fetal bovine serum. Before confocal observation, cells were washed with 3× PBS and fixed with 4% formaldehyde diluted in DI water at room temperature for 20 min. After that, 0.1 mL of Triton X was added for 30 min under room temperature. Cells were blocked for nonspecific binding using 1% BSA and incubating at 37 °C for 5 min. After blocking, the primary antibody (Anti-Vinculin, mouse from Sigma Merck, UK) was added for 12 h under 4 °C. Alexa Fluor 594 Phalloidin, 1:300 (from Thermo Fisher, UK), and Hoechst 33258 (from Thermo Fisher, UK) were applied at room temperature for 1 h. Cells were next washed with 0.5% Tween in PBS three times. Then, a biotinylated secondary antibody (mouse, UK) was added and incubated at room temperature for 1 h.

### 2.7 Estimated FN Charge

The charge of FN strongly affects Coulomb’s force between the FN and nanomaterials. FN is a glycoprotein of the ECM that is coded by the FN1 gene. The plasma FN has two nearly identical polypeptide chains connected by two disulfide bonds present near the carboxy terminal. Each polypeptide chain is nearly 250 kDa. The sequence we used for calculating the charge is isoform1 (Uniprot KB-P02751), with the length of 2386 and mass of 262 kDa. Protein calculator v3.4 was used for calculating the charge of FN based on its monomer sequence. During the calculation, pH = 7 was set as the condition where the protein is in, consistent with our experiment conditions. The charge of FN was estimated by protein calculator based on the hypothesis. The hypotheses were as follows;

1 Molecular weight of FN was 440 kDa with an unfold structure.
2 All residues have p*K*_a_ values that are equivalent to the isolated residues.
3 The p*K*_a_ values for the individual amino acids were from Stryer Biochemistry, 3rd edition.

## 3 Results

### 3.1 Nanotopographies with Controlled Curvature on Subunit Features

**Figure 1:**
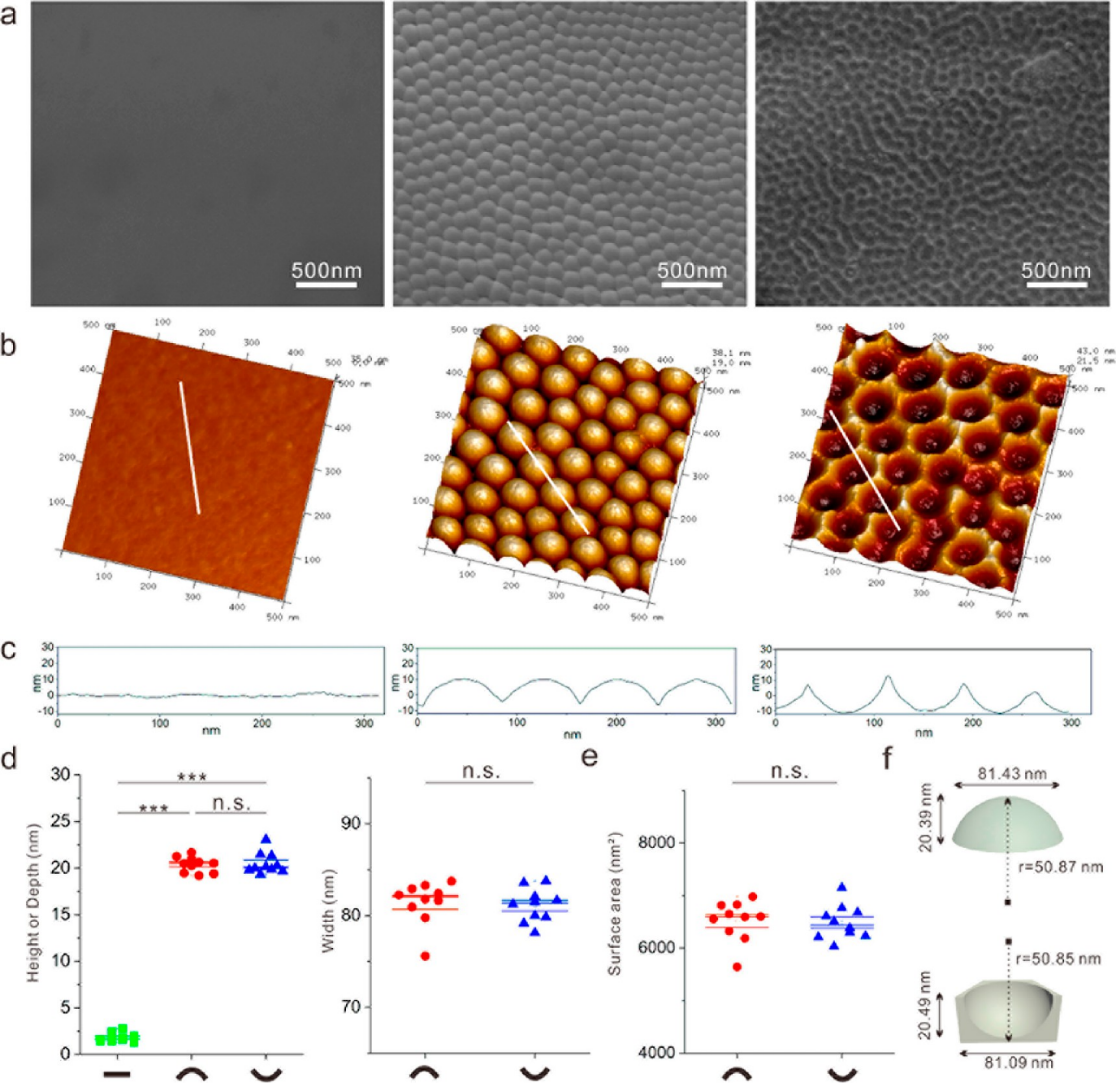
Topographical characterization of nanoflat, nanoconvex, and nanoconcave. (a) Topography of nanoflat, nanoconvex, and nanoconcave by SEM, illustrates the nano scale flat of nanoflat, and highly ordered arrangement of subunits on nanoconvex and nanoconcave. (b) Three-dimensional illustration of nanoflat, nanoconvex, and nanoconcave by AFM tapping mode in the 500 × 500 nm^2^ square area. (c) Sectional dimensions of three topographies. (d) Based on the AFM section measurement, the statistic dimensions of nanoflat (height), each subunit (height and width) of nanoconcave and nanoconcave (height and width). Ten subunits on different nanotopographies were analyzed (*n* = 10). ***, *p* < 0.001; n.s., not significant. Error bars represent the standard error with the mean (s.e.m). (e) Surface area of nanoconvex and nanoconcave. Nanoconvex and nanoconcave are seen as a spherical dome. (f) Schematic illustration of dimensions of nanoconvex and nanoconcave in average, respectively.

A first major challenge was to create TiO_2_ nanotopographies with identical curvature/high fidelity of the nanofeatures and similar Coulomb’s force generated by each nanofeature. Coulomb’s force relies on the electric charge produced by both the protein and subunit, and the separation distance. In terms of Ti material surface charge, the electrons are prone to transfer to adjacent regions of higher curvature when FN adsorption, leading to an increased charge density at this region.^33^ Thus, the high curvature region generates enhanced Coulomb’s force compared to regions of low curvature. Figure 1 shows the topographical characteristics of the nanoflat, nanoconvex, and nanoconcave surfaces. Nanoconvex and nanoconcave surfaces exhibited symmetrical topographies; nanoconvex consisting of spherical caps, and nanoconcave consisting of an egg-box pattern. The arrangement of nanofeatures on the nanoconvex and nanoconcave surfaces was highly organized, as shown in Figure 1a,b. Figure 1c demonstrates that the curvatures of both nanoconvex and nanoconcave subunits were identical at the nanoscale. Nanoflat morphology had a nanometric mirror finish with 1.79 nm average height/depth. Ten nanofeatures on the nanoconvex and nanoconcave surfaces were selected for statistical measurement, as illustrated in Figure 1d–f.

### 3.2 Surface Chemical Composition of Nanotopographies

**Figure 2:**
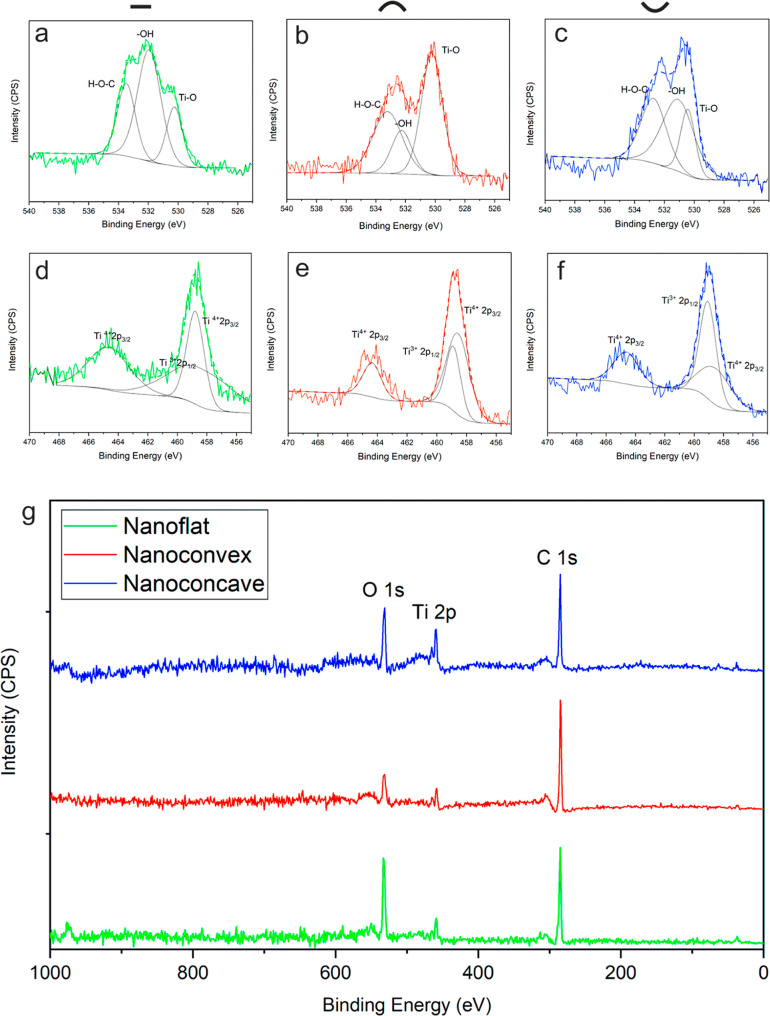
Chemical composition analysis by XPS. (a) High-resolution scan of O1s of nanoflat. (b) High-resolution scan of O 1s of nanoconvex. (c) High-resolution scan of O 1s of nanoconcave. (d) High-resolution scan of Ti 2p of nanoflat. (e) High-resolution scan of Ti 2p of nanoconvex. (f) High-resolution scan of Ti 2p of nanoconcave. (g) Full spectra scan of nanotopographies.

Materials chemistry also plays a central role to manipulate cell responses, and thus, the chemical characterization of nanotopographies is an initial step but necessary for further proving the influence of topographical effects. The chemical composition of nanotopographies was characterized by XPS. In specific, of oxygen (O 1s) peak (Figure 2a–c), titanium (Ti 2p) peak (Figure 2d–f) on nanotopographies were scanned by high-resolution spectra. Meanwhile, the chemical composition was measured by the full spectrum scan. From Figure 2g, the nanoflat, nanoconvex, and nanoconcave have shown three main peaks of O 1s, Ti 2p, and C 1s, and this indicates that the compositions of all topographies were identical and consist with TiO_2_, which is the main composition formed by the natural oxidation of Ti-based orthopaedic implants.

### 3.3 Surface Potential of Nanotopographies

**Figure 3:**
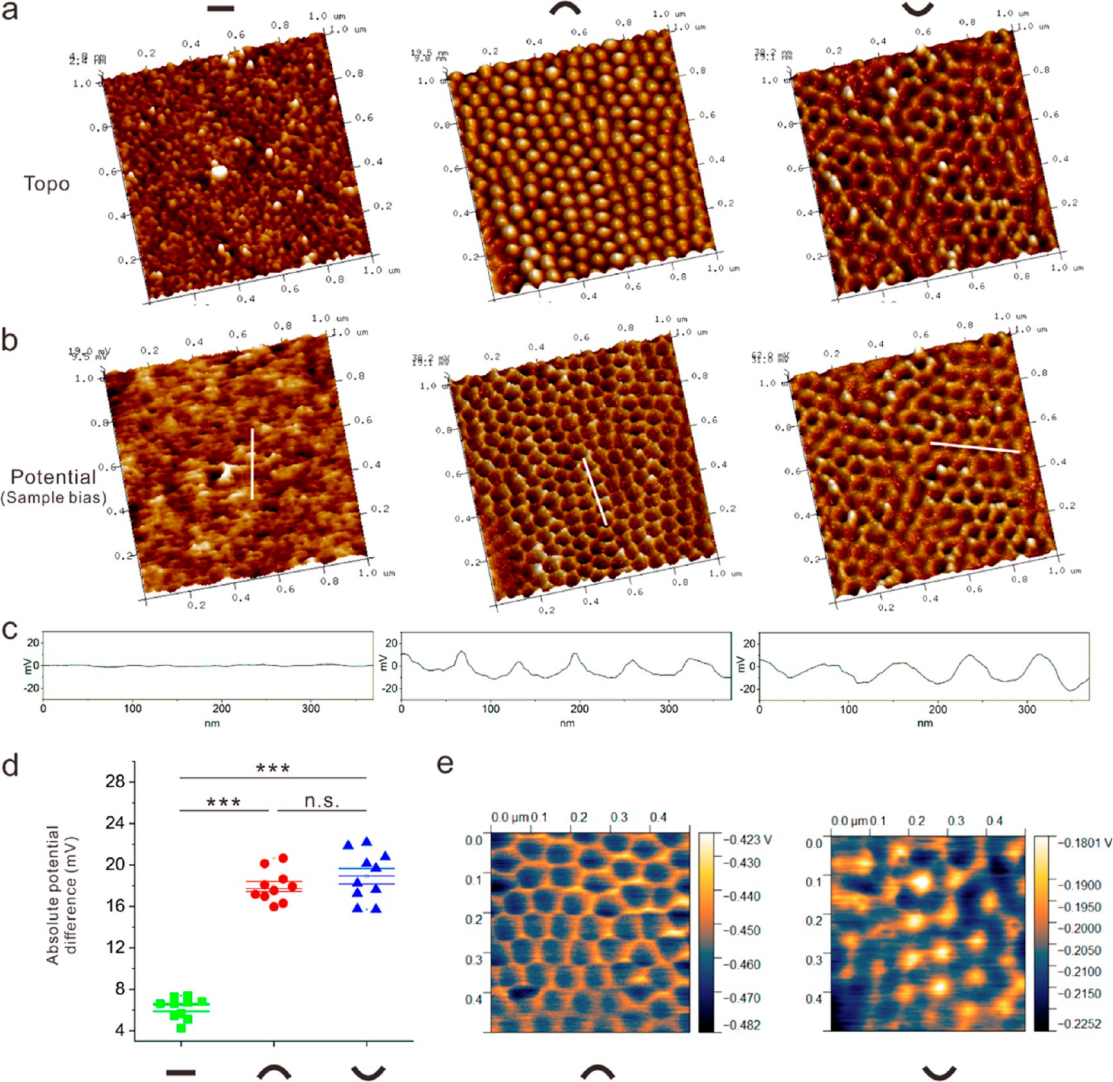
Relative surface potential distribution on nanoflat, nanoconvex, and nanoconcave. (a) AFM topographical characterization of nanoflat, nanoconvex, and nanoconcave by the tapping mode in 1 × 1 μm^2^ square area. (b) Surface potential distribution measured by KPFM with the sample bias model in identical topography 1 × 1 μm^2^ square area. The surface potential of each topography displays highly correlated with the topographical features. With the sample bias model, topographical bumps on nanoconvex are shown bowls in potential distribution, and nanoconcave displays a constant shape in both topography and surface potential distribution. (c) Sectional potential distribution in relative value on nanoflat, nanoconvex, and nanoconcave. (d) Absolute potential difference on each subunit of nanoflat, nanoconvex, and nanoconcave. Ten subunits on different nanotopographies were analyzed (*n* = 10). ***, *p* < 0.001; n.s., not significant. Error bars represent standard error compared with the mean (s.e.m). (e) Measurement of surface potential of nanoconvex and nanoconcave calibrated by HOPG.

Because electron motion is regulated by the topographical curvature, we then measured the surface potential of the nanotopographies using KPFM. The corresponding features between surface potential and topographies in the absence of an external electric field are illustrated in Figure 3a,b. The surface potential difference of the nanoflat surfaces exhibited the highest consistent, this is due to the mirror planarity down to the nanoscale. The nanoflat surface was designed as a control featured with the uniform distribution of electrons. Interestingly, the potential difference between the nanoconvex and nanoconcave surfaces had the same trend, where the top of a convex feature or valley of a concave feature exhibited higher potential for each subunit (Figure 3b,c). This indicates that electrons on convex features distributed at the tip have a higher density and are “prone” to transfer to the bottom of the feature (B), and concave feature associated electrons are “prone” to transfer from the valley to the ridge. Furthermore, absolute potential difference (calibrated by HOPG) of topographical subunits of nanoconvex and nanoconcave surfaces have no statistical difference, and are significantly higher than on the nanoflat surfaces (Figure 3d,e).

The dimension of FN is strongly dependent on its conformation. In general, FN could be visualized as two identical strands with 61 nm in length, with a molecular weight of 440 kDa.^36,37^ At the nanoscale, because the charged FN attraction is influenced by the adjacent charge on a topographical subunit (size, 80 nm), the anchoring points are possibly the strongest attractive region on the subunit. It is recognized that cells do not directly interact with material surfaces, but cell interaction with a material surface depends on the ligand density of ECM for anchorage. However, because the FN is adsorbed at the cell nanotopography interface, the direct determination of adsorbed FN at the cell–matrix interface is unfeasible. Instead, a method is accomplished to speculate FN arrangement though analyze cellular adhesion dimensions, as the high dense of FN can stimulate the maturement of the FA complex.

### 3.4 HOb Initial Adhesion on Nanotopographies

**Figure 4:**
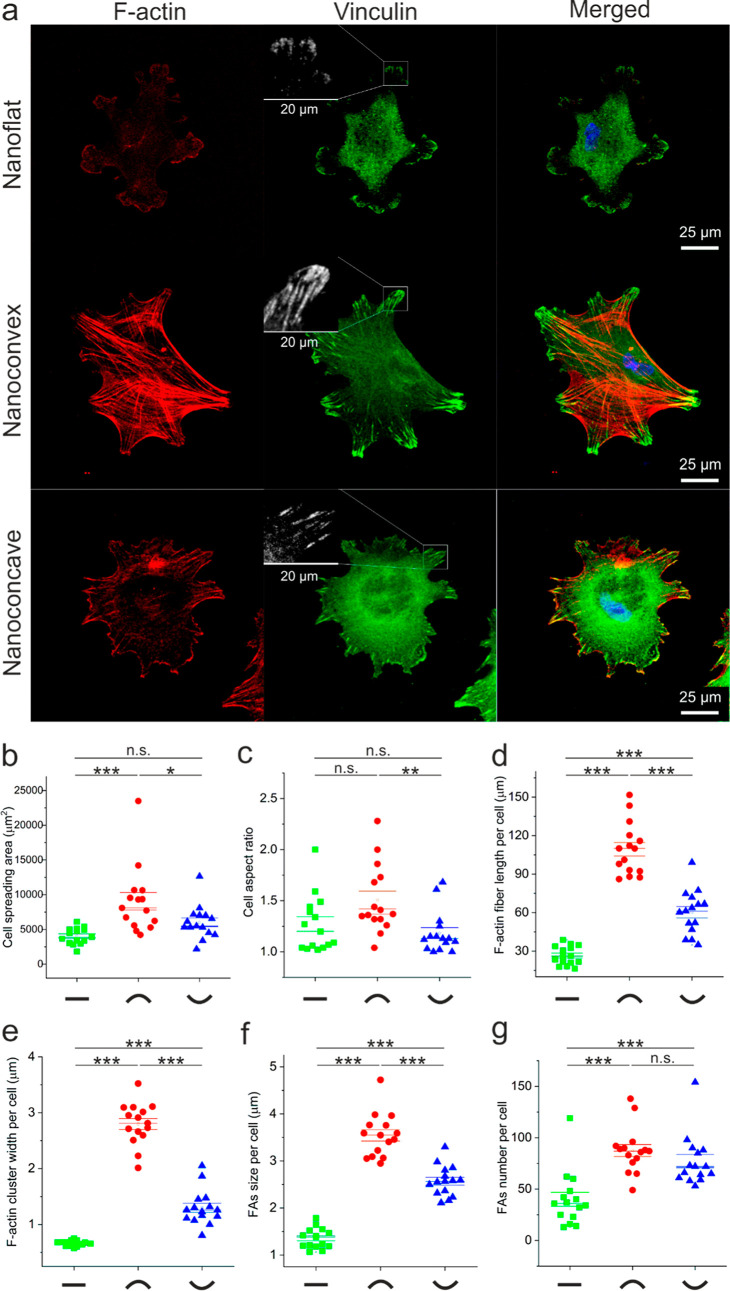
F-actin and FA quantification. (a) HObs after 3 h on FN adsorbed nanoflat, nanoconvex, and nanoconcave. First column shows F-actin cytoskeleton arrangement, second one is FA plaques (vinculin), and third one is merged image. (b) Cell spreading area measurement. (c) Aspect ratio measurement per cell. (d) The length of F-actin per cell. (e) F-actin cluster width per cell. (f) FA size per cell. (g) Focal adhesion quantification per cell. 15 cells on different nanotopographies were analyzed (*n* = 15). ***, *p* < 0.001; **, *p* < 0.01; *, *p* < 0.05; n.s., not significant.

Cells’ initial response (3 h) to nanotopographies was first analyzed by cell morphological quantification. Figure 4a shows the HOb initial attachment on nanotopographies coated with 5 μg/mL FN. HObs were seeded at a low density 1200 cells/cm^2^ to allow focus on cell–material interactions. In particular, cells had a similar size on both the nanoconcave and nanoflat; however, the cell size was significantly larger on the nanoconvex (Figure 4b). As the HObs have a spindle morphology, the cell aspect ratio is also a factor to consider. Cells on the nanoconvex surfaces had the highest aspect ratio value, indicating osteoblasts were more elongated than those on the nanoconcave and nanoflat surfaces. However, no significant difference of the aspect ratio was observed by the statistical analysis between nanoflat and nanoconvex. The elongated cellular morphology is associated with cytoskeleton tension and FA characters. F-actin immunostaining demonstrated the well-organized F-actin architecture of cells both on nanoconvex and nanoconcave features. However, the dimensional features of stress fibers in cells on nanoconvex, width and length, were significantly increased than those on nanoflat and nanoconcave surfaces (Figure 4d,e). Cytoskeleton microfilaments are tethered to integrins. These transmembrane receptors are recruited and clustered into groups due to the interaction between ECM and integrins.^38^ The integrin binding complexes along with the adhesome mechanically couple integrins to the force generating actomyosin system, acting as a “molecular clutch” to transfer load stimuli.^39^ Thus, FA formation can directly impact on cytoskeleton tension, thus directly affecting mechanotransductive pathways. FA maturation levels are thus important to cell adhesion to the material surface and subsequent cellular response.

FA complexes analysis was based on vinculin-stained confocal images; note that dot FAs shorter than 1 μm were discarded. In cells on nanoconvex surfaces, supermature FA (>5 μm in length) and FA length in the range of 3–4 μm were observed. However, the length of FA was significantly shorter in cells on nanoconcave (2–3 μm in length) and nanoflat (1–2 μm in length, Figure 4f). However, the quantitative analysis of FA in cells on nanoconvex and nanoconcave surfaces had no significance difference but was higher than for FA number on nanoflat samples. Integrin activation at the plasma membrane is followed by a force transduction and changes in actin/biochemical regulation.^40^ Considering the integrin interacts with RGD ligands in FN, assembly, anchorage, and integrin clustering is strongly influenced by ligand rigidity and distribution in nanoscale. Because the nanotopographies consist with TiO_2_, it is hypothesized that the rigidity of the TiO_2_ layer on nanotopographies has a similar modulus. It can thus be proposed that the adhesion-mediated interactions are regulated by the spatial arrangement of the ECM regulated by the nanotopographical features. Previous study has demonstrated the FA in cells was restricted mainly to the periphery of cells growing on 108 nm RGD spacing nanopattern, whereas cells on 58 nm patterns displayed numerous mature FAs.^41^ While the separation of adhesive dots by more than 73 nm results in the limitation of cell attachment, a ligand range of 58–73 nm is an universal length scale for integrin clustering and activation.^42^

For FN–TiO_2_ dynamic interactions, the FN spacing and organization is influenced by the electrostatic adsorption process. However, electrostatic adsorption is undetectable due to the in situ situation and interference of charge when inducing a test probe. The formation of mature FA in cells on nanoconvex surfaces indicates that integrin clusters form with a “precisely tailored” spacing of FN at the nanoscale which is adsorbed on the surface. Thus, the dominant electrostatic attraction-Coulomb’s force was evaluated by simulation methodology.

### 3.5 Contact and Anchorage of Cells on Nanotopographies Analyzed by SEM

**Figure 5:**
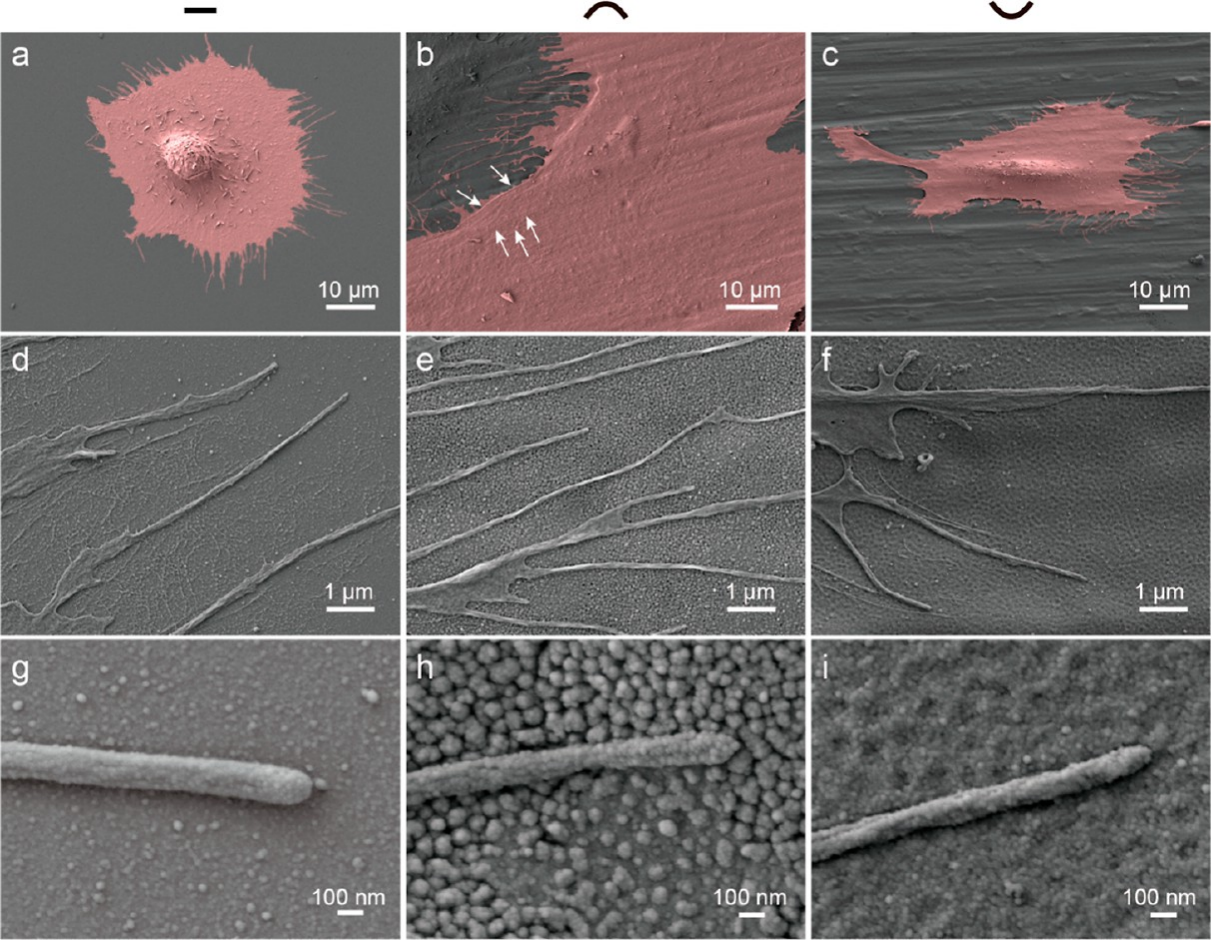
Cell morphological features by SEM. (a) HObs after 3 h on FN adsorbed nanoflat. (b) Cell morphological features on nanoconvex. (c) Cell morphological features on nanoconcave. (d) Filopodia extensions on nanoflat. (e) Filopodia extensions on nanoconvex. (f) Filopodia extensions on nanoconcave. (g) Filopodia features on nanoflat. (h) Filopodia features on nanoconvex. (i) Filopodia features on nanoconcave.

Cell cytoskeletal features and cell nanotopographies interactions were observed that by SEM. Cells on nanoflat were spherical and poorly spread, the nucleolus appeared thicker, but flattened in the peripheral regions. The cell membrane was also shown limited protrusion (Figure 5a). Osteoblasts’ membrane on nanoconvex was exhibited thin and fully flattened appearance, and thus, the stress fibers in the cytoskeleton were visible (white arrows in Figure 5b). Cells on nanoconvex were formed massive of filopodia extending, protruding filopodia was associated with the initial spreading behavior of cells (Figure 5e). Filopodia contain receptors for diverse signaling molecules and ECM molecules, integrins, and cadherins are often found in the tips of filopodia, and thus, the integrins accumulate in filopodia are primed to probe the matrix, creating “sticky fingers” along the leading edge that promotes cell adhesion.^43^ Osteoblasts on nanoconcave were seen with reduced spreading, and the nucleolus was thicker than the membrane protrusion (Figure 5c). The filopodia nanotopography interactions are shown in Figure 5d–i, and the filopodia on cells were fully contacted and interacted with the nanotopographies.

### 3.6 Modeling Coulomb’s Force Evolution on Nanoconvex and Nanoconcave Surfaces

The Coulomb’s force is proportional to the charge of both FN and nanotopography and inversely proportional to the square of distance between the two charges. The actual charge of FN is complex and differs substantially due to several factors, such as the conformation, solvent concentration, bonding ions, pH, dielectric constant, and temperature in the molecule scale.^44,45^ In nanoscale, in order to investigate electrons motion on nanotopographies, the structure of FN was simplified to a point charge, and one of the hypotheses is that the charge of FN does not change during adsorption on the surfaces. The detailed charge of FN is estimated and illustrated in the methodology section. However, the charge of the nanoconvex and nanoconcave surfaces is dynamic with electron transferal due to the relative location between FN and nanotopography. Electrons can only transfer on the surface of the material, indicating that surface features, such as curvature, play dominant roles in dynamic charge. Thus, a three-dimensional morphological model was established for the topographical features of the nanoconvex and nanoconcave surfaces. The boundary of charge density distribution is determined by experimental results obtained by KPFM under equilibrium (non-inducement of external charge, such as FN). Meanwhile, because both nanotopographies are fabricated in TiO_2_, it was assumed that the charge density on nanotopographies is homogeneous without the inducement of external FN. In order to investigate the electron motion on nanotopographies, a nano-subunit can be mathematically differential into unlimited “diminutive areas” which can be defined as electrons on different locations on the surface (detailed modeling procedure in Supporting Information, Figure S5). Thus, the charge density of a nanosubunit can be described as the integral of all “diminutive areas”.

**Figure 6:**
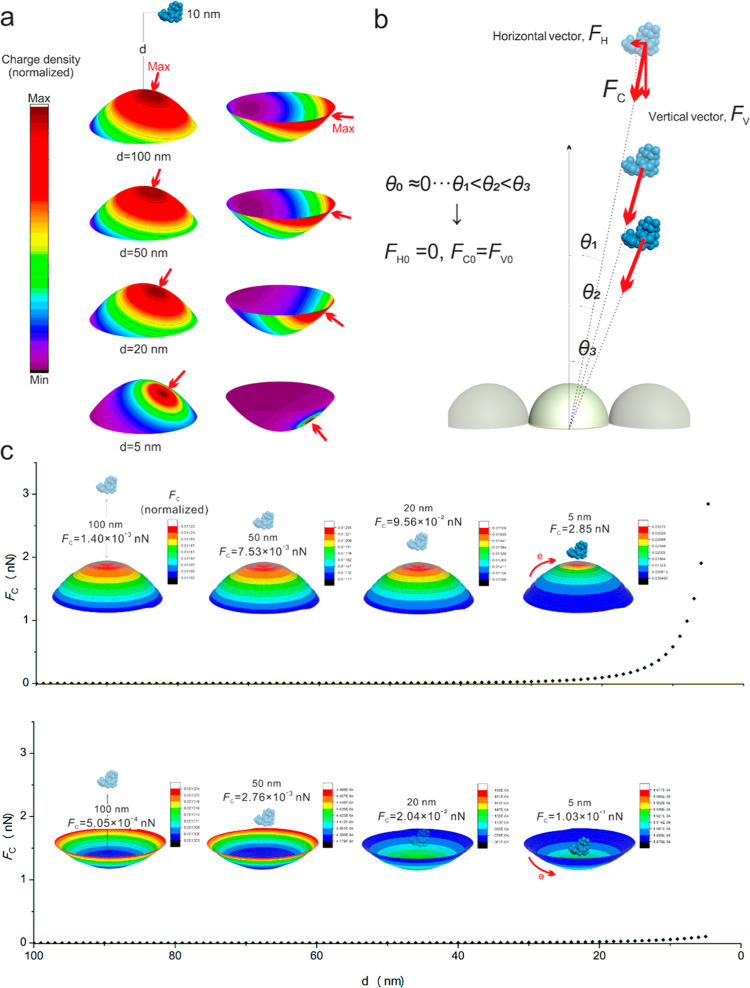
Charge density evolution on nanoconvex and nanoconcave of FN adsorption. (a) Distribution of charge density and maximum charge areas while FN from 100 to 5 nm with excursion of 10 nm. (b) Schematic illustration of Coulomb’s force (*F*_C_), horizontal (*F*_H_), and vertical (*F*_V_) vector. (c) Variation of Coulomb’s force (*F*_C_) and the distance between FN and nanoconvex/nanoconcave. The *F*_C_ of nanoconvex increased dramatically with the FN attracted by the surface from 100 to 5 nm, and the maximum of *F*_C_ reaches to 2.85 nN at *d* = 5 nm.

Figure 6a illustrates situations for the evolution of charge density when FN adsorbs onto nanoconvex and nanoconcave surfaces from 100, 50, 20, and 5 nm distances and with a 10 nm offset. The maximum density of charge is at the region adjacent to the tip area on the nanoconvex surfaces and the ridge area of the nanoconcave surfaces. The maximum charge density on both the nanoconvex and nanoconcave surfaces is prone to be “off-center” with the increase of *d*. The phenomenon is described in Figure 6b. Coulomb’s force (*F*_C_) can be divided into horizontal (*F*_H_) and vertical (*F*_V_) directions, respectively. The attraction between FN and the nanofeature is initiated from the infinite large distance between the FN and nano-subunit, the angle (defined as θ) between the line connects FN and the subunit and the vertical line of the subunit is approximately equal to 0. At the initial stage, the contribution of *F*_H0_ is infinitesimal compared with *F*_V0_, Coulomb’s force (*F*_C0_) is also approximately equal to the vertical (*F*_V0_) vector. It is indicated that the initial Coulomb’s force is mainly contributed by the vertical vector. Thus, to further reveal the evolution mechanisms, we investigated the situation of FN vertically adsorbing on to nanosubunits of both nanoconvex and nanoconcave surfaces.

In the case of FN is adsorbing from along the central line of the subunit, it is only subject to the vertical vector, *F*_V_ = *F*_C_. As shown in Figure 6c, the Coulomb’s force evolution of nanoconvex and nanoconcave features exhibited obvious differential behaviors. The *F*_C_ of nanoconvex features roughly threefold higher than that of nanoconcave when FN attracted at a location from 100 to 50 nm toward the nano-subunit center. Meanwhile, the high density region of charge is at the tip region on nanoconvex features and the ridge region on nanoconcave features. However, as the distance changes to below 20 nm, the *F*_C_ of nanoconvex features significantly increases, and the area of attraction region on tip of the feature is also decreased. Interestingly, the main attractive region of nanoconcave features transferred to the bottom area as the FN adsorbs. When the distance from FN to the nanotopographical subunit is 5 nm, the *F*_C_ of nanoconvex features reaches 2.85 nN, which is 30 folds greater than that of nanoconcave features (1.03 × 10^–1^ nN). The comparatively Coulomb’s attraction to FN between nanoconvex and nanoconcave was further analyzed. To achieve this, each one of subunit on nanoconvex and nanoconcave was selected to simulate the redistribution of charge density at different regions on the subunit.

**Figure 7:**
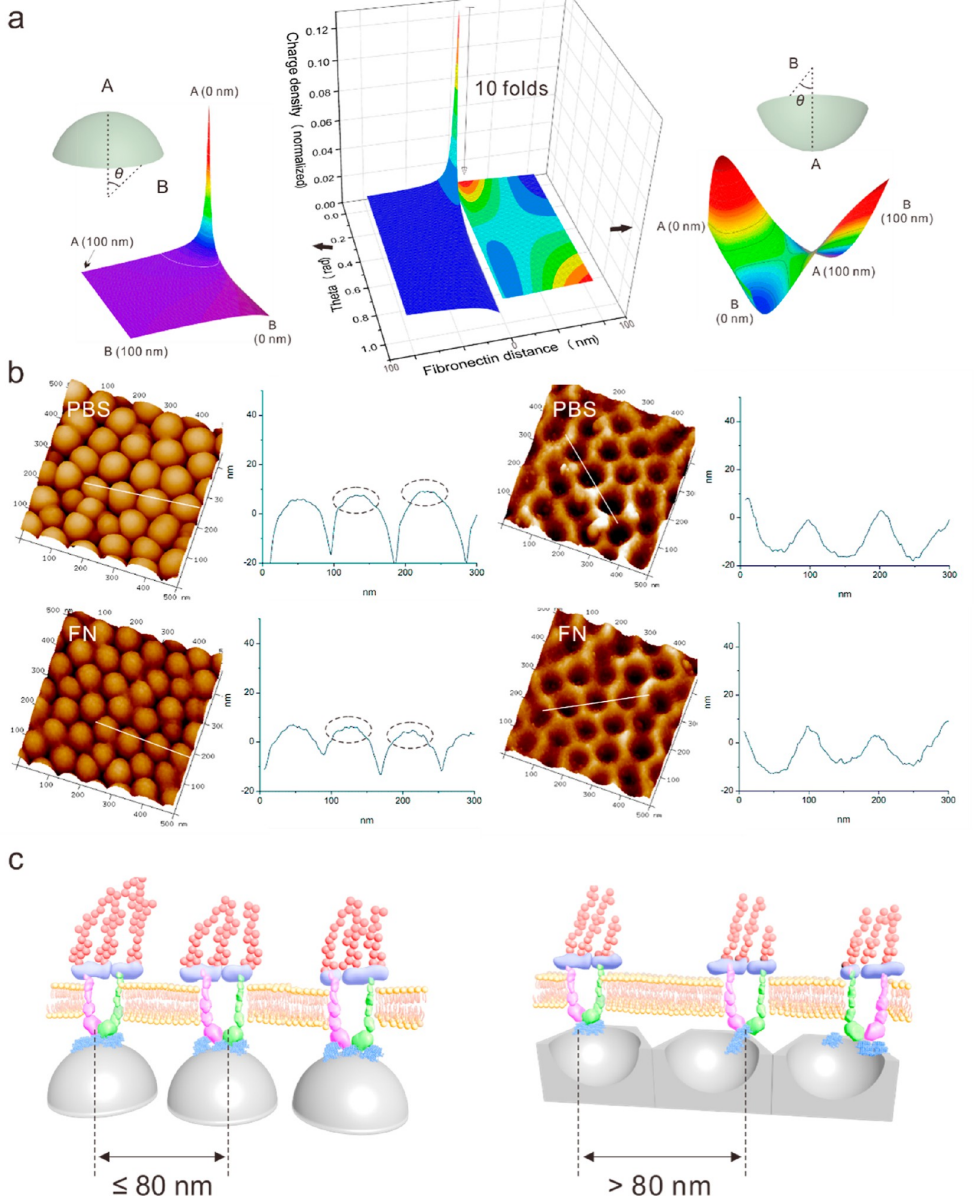
Simulation and experimental investigation of FN–subunit interactions. (a) Dynamic map of the charge density of regions on a subunit of nanoconvex and nanoconcave when FN is adsorbing from 100 to 0 nm. When FN is attracted to the subunit, the charge density on nanoconvex top (A) is greatly increased, the charge density on ridge (B) is slightly decreased. Charge density on nanoconcave bottom region (A) is increased in the range from 100 to 20 nm, and decreased from 20 to 0 nm, charge density on ridge (B) is decreased from 100 to 5 nm, and slightly increased from 5 to 0 nm. (b) In situ topographical characterization of nanoconvex and nanoconcave in PBS solution and in FN solution. Sectional dimension has shown that the FN is mainly adsorbed at the top region on the nanoconvex, and the FN was distributed homogeneously on the nanoconcave. (c) Illustration of the FN adsorptive distribution of nanoconvex and nanoconcave, the high dense distribution (<80 nm) could increase the ligand density and form matured FA, the low density of distribution (>80 nm) forms dot FA.

Evolution of Coulomb’s force, *F*_C_ of nanoconvex and nanoconcave features has demonstrated differential electron migration. Due to the topographical features, electrons are consistently transferred to the tip region (Figure 7a—point A) of nanoconvex features, leading to a sharp increase of charge density. Because the electrons are prone to concentrate at point A, the other areas on the nanoconvex samples, such as ridge area B where electron density was significantly decreased, resulting in a significantly different charge density on nanoconvex features. Compared with nanoconvex surfaces, the nanoconcave surfaces have demonstrated a complex Coulomb’s force evolution. Electrons first migrate to the ridge region (Figure 7a—point B), leading to the maximum charge density. However, when the FN is attracted to a distance of 50 nm from the nanofeature subunit, the electrons transfer toward the bottom of the nanoconcave features (Figure 7a—point A). When the distance between FN and nanoconcave features is less than 50 nm, the electrons migrate to point A. The inconsistent migration of electrons on the nanoconcave features exhibits a “saddle shape” of charge density distribution (Figure 7a). Importantly, the comparative analysis of the charge density map has shown that the maximum value of charge density on the nanoconvex features is 10-folds than that seen on the nanoconcave features. The minimum charge density of the nanoconvex features is at point B when FN is adsorbed at point A, where the value is slightly lower than the minimum charge density of nanoconcave features. Based on Coulomb’s force evolution, it can be speculated that the FN has high probability to be “captured” and constrained at point A of nanoconvex features by the intensive electrostatic attraction. Enhanced FN adsorbed at the top region leads to a high-density distribution in adjacent areas. Meanwhile, other regions of the nanoconvex features can only generate a weak attractive force because of limited deposition of FN. Compared with Coulomb’s evolution of the nanoconvex charge features, a more homogeneous charge density distribution is achieved on nanoconcave features caused by inconsistent electron migration. The FN absorbance is interfered by various transfers of electrons, having a more random and/or uniform distribution of FN than that on convex topographies. To demonstrate our hypothesis, the 3D view of nanoconvex and nanoconcave features with adsorbed FN, compared to the nanofeatures without FN in PBS, were characterized using tapping mode AFM (Figure 7b). The sectional profile of nanoconvex tip was obtained while measuring nanoconvex features in PBS, whereas an obvious amplitude profile was observed at the tip region in nanoconvex features incubated with FN/PBS solution. Numerous tiny bumps were observed at the tip of features in FN/PBS, and the edges of single subunit were clear. This indicates that the FN was barely arranged at the edge, on nanoconvex surfaces. In contrast, in the section views of the nanoconcave features in both PBS and FN/PBS differences were not obvious. Topographical fluctuations were observed on both the bottom and the ridge areas on subunits of nanoconcave topographies. Furthermore, the 3D reconstructed topography of nanoconcave features in PBS showed sharp edges and deep bottoms, whereas the number of both the edges and bottoms were decreased with FN/PBS. This indicates that the FN not only adsorbed on bottom of subunits but was also arranged at ridges.

### 3.7 Conformation of FN Adsorbed on TiO_2_ Nanotopographies

**Figure 8:**
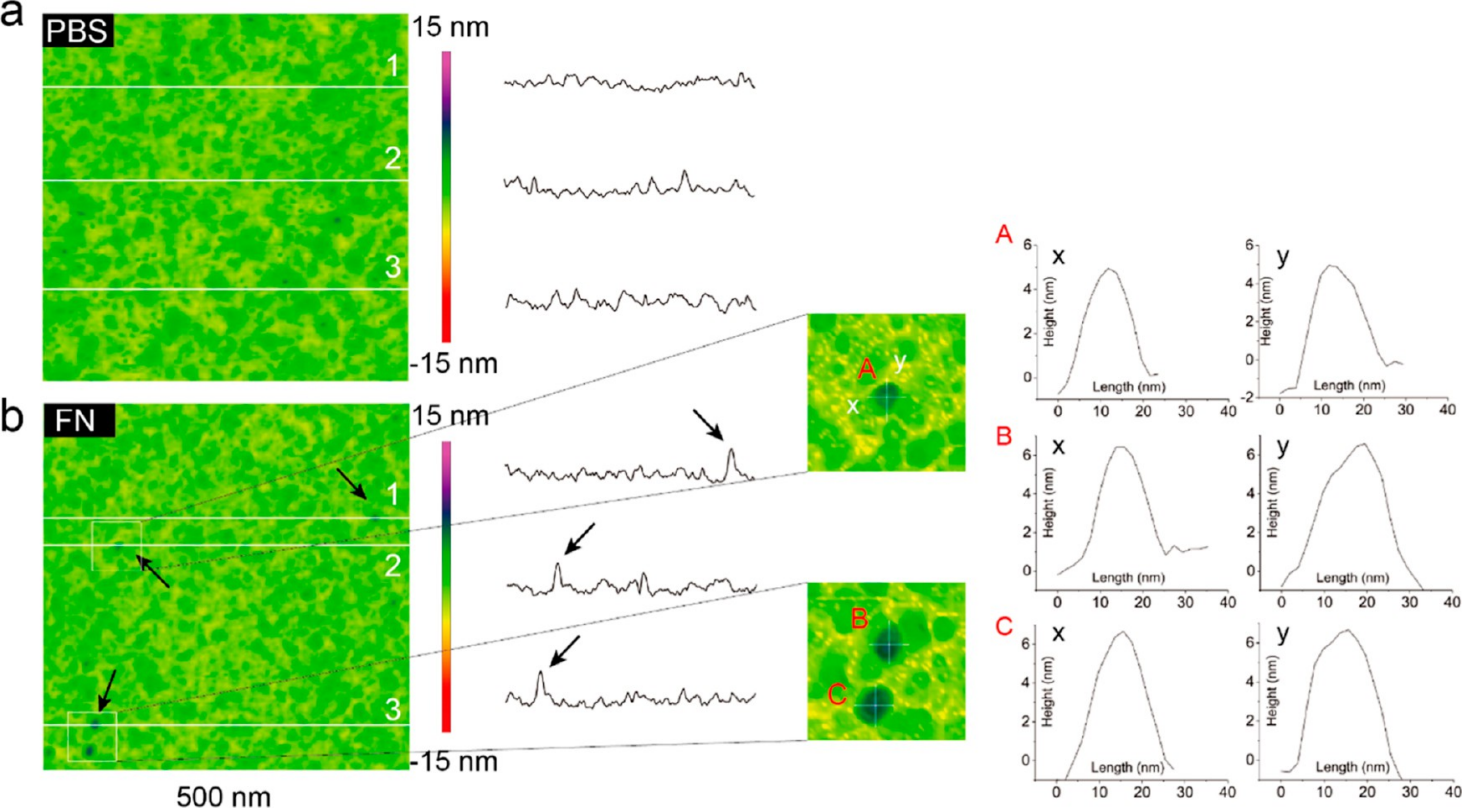
Morphological comparison analysis of nanoflat in PBS/FN and the conformation of FN adsorbed on nanoflat. (a) Topological characterization of nanoflat in PBS. (b) Topological characterization of nanoflat with adsorbed FN, the FN on nanoflat exhibited compact conformation. 1, 2, and 3 are the sectional tracks of morphological comparison between nanoflat in PBS/FN.

The morphological conformation of FN adsorption is the first challenge for further identification of the distribution of FN on nanoconvex and nanoconcave. In terms of identifying FN conformation on TiO_2_, the morphology comparative analysis of nanoflat in PBS and in FN was carried out. As shown in Figure 8, the morphological difference between nanoflat in PBS and in FN was the presence of FN, which featured with “globular particles” distributed on nanoflat in FN. Three FN particles were selected and sectional analyzed (Figure 8A–C). The FN molecules were featured in a round shape, 20 nm in diameter and 6–7 nm in height and with “compact” conformation. These dimensional features could be the morphological reference of FN adsorption on nanoconvex and nanoconcave.

**Figure 9:**
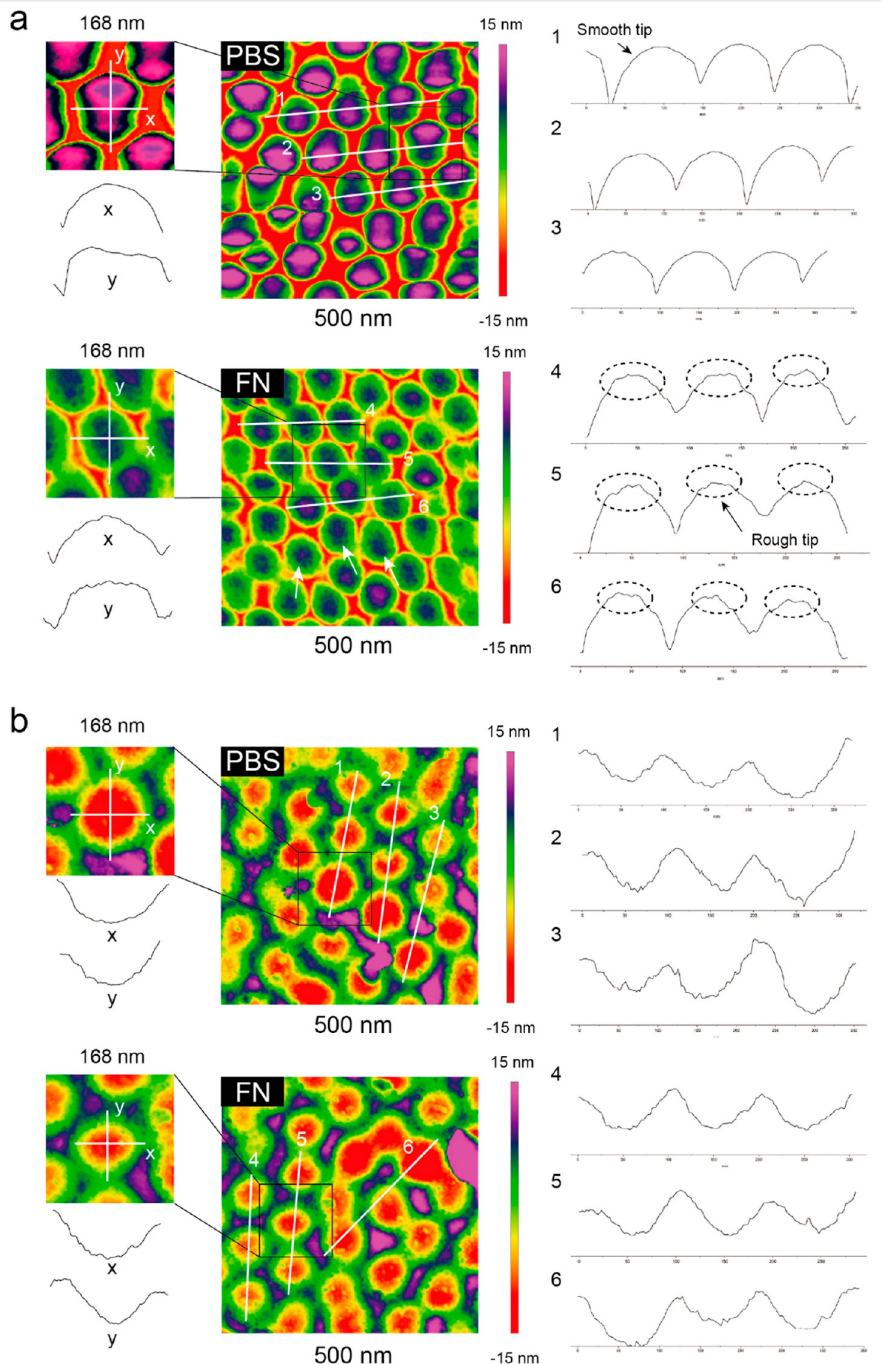
Morphological analysis of FN adsorption on nanoconvex and nanoconcave surfaces. (a) AFM imaging of FN on nanoconvex in PBS and FN, nanoconvex tip is smooth in PBS and rough in FN. (b) AFM imaging of FN on nanoconcave in PBS and FN, the morphology of different regions on concave have shown identical features.

The detailed morphological analysis of nanoconvex and nanoconcave is shown in Figure 9. The morphological features of “smooth tip” was observed on nanoconvex in PBS, see Figure 9a. In comparison, the tip on nanoconvex in FN has shown a “rough tip”, from the top view, and the tip region has a clustering of nanoparticle feature (Figure 9a, white arrows). The diameter of the region of “rough tip” was 32.88 nm in average (see Supporting Information, Figure S1). Meanwhile, the other region on nanoconvex in FN contains similarity features compared with in PBS. Figure 9b illustrates the morphological comparison of nanoconcave in both PBS and FN, containing the identical features, from the top and sectional view.

## 4 Discussion

In this study, we reveal the evolution of Coulomb’s force produced by FN–nanoflat, nanoconvex, and nanoconcave topographies, further instruct cell adhesion and spreading due to influence ligand spacing. A large spreading area and spindle-like cellular morphology was observed in cells on nanoconvex features. Meanwhile, mature FA and organized cytoskeleton large fibers were also found in cells on the convex topography. Our model has demonstrated that a high-density arrangement of FN is achieved at the tip area on subunits of nanoconvex features, as electrons are prone to migrate to tip regions generating an intense and consistent attraction to proteins.

High-density adsorbed FN interacts with numerous integrin ligands, which cluster and further activate the formation of large FA. The model also pointed out the homogeneous and/or random distribution of FN on nanoconcave features where a comparative low-density of FN was projected to be arranged on the subunits. As aforementioned, previous studies have demonstrated that the critical ligand separation length is 73 nm.^42^ An optimal ligand spacing of 60 nm has also been ascribed to the 60 nm dimensional features of talin, which is one of the cytoplasmic proteins acting as a crosslinker during integrin aggregation.^46,47^ The diameter of a nanoconvex subunit is 80 nm, and from the statistically morphological analysis, the FN concentrated region is <40 nm (Figure S1). It can be speculated that single or multiple FN are adsorbed at the tip which generates anchor points and further binds with integrin ligands. Numerous nanoconvex subunits can generate a high dense clustering of FN arrangement which thus bind with a high density of integrins. Moreover, integrins aggregate and might further “activate” talin and trigger FA gathering and cytoskeleton formation/contraction. Due to the identical charge of FN, electrostatic repulsive force between FN–FN generates a minimum ligand spacing. The minimum ligand spacing may also be affected by the distance between integrins in the cell membrane which is around 8–12 nm.^48^ Thus, the ligand distribution on nanoconvex features is illustrated in Figure 7c. In contrast, a more homogeneous and random distribution of FN creates a low-density of anchor points to bind with integrin ligands which impairs the downstream molecule conjugation. Thus, a less conjugated cytoskeleton in cells on nanoconcave was obtained. The weak cellular adhesion on nanoflat can be speculated due to the nanoscale of the planar surface, and the electrons are distributed homogeneous, leading to a weak “capture” of FN. The low-density distribution of FN resulted a lacking anchoring of integrins and thus generated minimum both quantitative and qualitative FA in cells.

## 5 Conclusions

This work inspires the designing of an Ti-based implant with nanotopographies to instruct cellular behaviors through revealing unknown mechanisms of Coulombs’ force evolution. With the fabrication of nanoconvex and nanoconcave surfaces, both featured with identical curvature on nano-subunits, a model of investigating Coulombs’ force evolution between FN and nanotopographies was established. The “convex” nanotopographical features can generate a constant and more intense attractive force (around 30 folds higher than nanoconcave) to biomolecules, whereas the “concave” surface generates a mild and mutative attraction. Through the differential of Coulomb’s force evolution, the differential FN distribution was achieved, thus obtaining differential cell adhesion and spreading behaviors. This approach can be generalized to guide the strategy of generating nanotopography in a customized shape to affect cell behavior and also be used to develop new nanotopography surfaces in orthopaedic implantations.

## Supporting Information Available

Hypothesis and calculation information about the established Coulomb’s force modeling and static measurement of the diameter of “rough tip” on nanoconvex features in FN (PDF)
