# Supplementary material for: TiO2 Nanotopography-Driven Osteoblast Adhesion through Coulomb’s Force Evolution
Source: ACS Appl Mater Interfaces. 2022 Jul 22;14(30):34400–14. doi: 10.1021/acsami.2c07652 (PMC9354007; doi:10.1021/acsami.2c07652)
Supplement: Supplementary file 1 — am2c07652_si_001.pdf [file am2c07652_si_001.pdf]

Supporting Information

TiO_2 Nanotopographies-Driven Osteoblasts Adhesion through Coulomb's Force Evolution

Jiajun Luo[1,7], Shudong Zhao[1,2], Xiangsheng Gao[1,3], Swastina Nath Varma[1], Wei Xu[1,4], Maryam Tamaddon[1], Richard Thorogate[5], Haoran Yu[6], Xin Lu[4], Manuel Salmeron-Sanchez[7], Chaozong Liu[1]*,*

[1] Division of Surgery & Interventional Science, University College London, Royal National Orthopaedic Hospital, Stanmore HA7 4LP, United Kingdom

[2] Key Laboratory for Biomechanics and Mechanobiology of Ministry of Education, Beijing Advanced Innovation Center for Biomedical Engineering, School of Biological Science and Medical Engineering, Beihang University, Beijing 100083, China

[3] Beijing Key Laboratory of Advanced Manufacturing Technology, Faculty of Materials and Manufacturing, Beijing University of Technology, Beijing 100124, China

[4] Beijing Advanced Innovation Center for Materials Genome Engineering, Institute for Advanced Materials and Technology, State Key Laboratory for Advanced Metals and Materials, University of Science and Technology Beijing, Beijing 100083, China

[5] London Centre for Nanotechnology, University College London, London WC1H 0AH, United Kingdom

[6] Institute of Bioengineering, College of Chemical and Biological Engineering, Hangzhou Global Scientific and Technological Innovation Center, Zhejiang University, Hangzhou 310027, China

[7] Centre for the Cellular Microenvironment, University of Glasgow, Glasgow G12 8LT UK

*Corresponding authors:

Prof. Xin Lu, luxin@ustb.edu.cn

Prof. Chaozong Liu, chaozong.liu@ucl.ac.uk

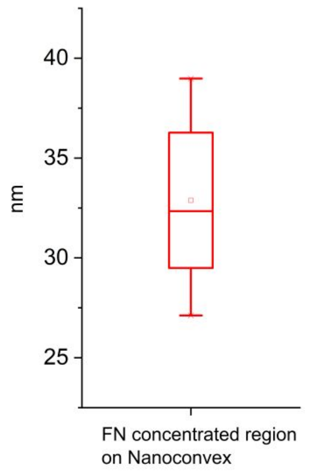

Figure S1. The static measurement of diameter of ‘rough tip’ on nanoconvex features in FN. Ten subunits on nanoconvex were analyzed (n=15), the average diameter is 32.88 nm.

Modelling was built based on hypothesis below:

Nanoconvex case;

The electron distribution can be divided into three status:

1. The equilibrium of single nanoconvex without external charged protein, the electrons are distributed uniform.
2. The adjacent charged protein causes the movement of electrons on nanoconvex surface.
3. Electrons re-equilibrium distribution on surface.

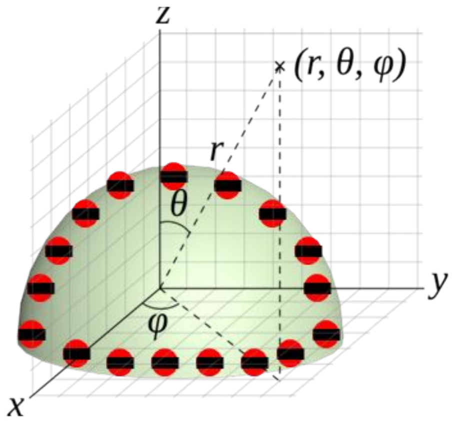

Equilibrium 1.

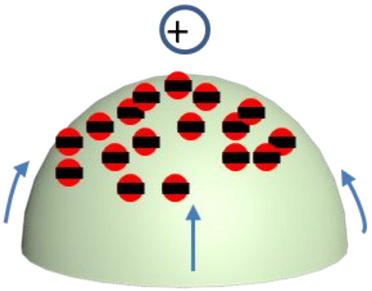

Equilibrium 2.

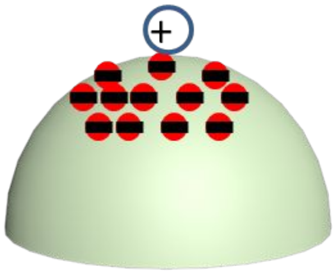

Equilibrium 3.

For equilibrium 1:

To comparative analysis the Coulomb's force of areas on nanoconvex, the general mathematically solution of the Laplace equation in spherical coordinates r and θ:

$$\nabla^2\varphi = \frac{1}{r^2}\frac{\partial}{\partial r}\left(r^2\frac{\partial\varphi}{\partial r}\right) + \frac{1}{r^2 sin\,\theta}\frac{\partial}{\partial\theta}\left(sin\,\theta\frac{\partial\varphi}{\partial\theta}\right) + \frac{1}{r^2 sin^2\,\theta}\frac{\partial^2\varphi}{\partial\emptyset^2} = 0$$

attempt of variables by writing,

$$\varphi(r, \Theta, \Phi) = R(r)\Theta(\theta)\Phi(\phi)$$

Then

$$R(r) = A_n r^n + B_n\frac{1}{r^{n+1}}$$

$$\Phi(\phi) = C_m sin\,(m\varphi) + D_m cos\,(m\varphi)$$

$$\Theta(\theta) = kP_n^m(cos\,\theta)$$

The single nanoconvex is symmetry by ϕ,

Then φ is related with r, θ,

$$\varphi(r, \theta) = \sum_n (a_n r^n + b_n\frac{1}{r^{n+1}})P_n(cos\,\theta)$$

Where, $P_n(cos\,\theta)$ is Legendre equation,

$$P_0(cos\,\theta) = 1,$$

$$P_1(cos\,\theta) = cos\,\theta,$$

$$P_2(cos\,\theta) = \frac{1}{2}(3cos^2\,\theta - 1)$$

$$\dots$$

Here, P_0 and P_1 were taken into account for the contribution of $\varphi(r, \Theta)$,

$$\varphi(r, \theta) = a_0 + \frac{b_0}{r} + (a_1 r + \frac{b_1}{r^2})\cos\theta$$

when $r = \infty$, $\theta = 0$, $\varphi(\infty, 0) = 0$, then

$$\varphi(r, \theta) = \frac{b_0}{r} + \frac{b_1}{r^2}\cos\theta$$

where, b_0 and b_1 are undetermined coefficients.

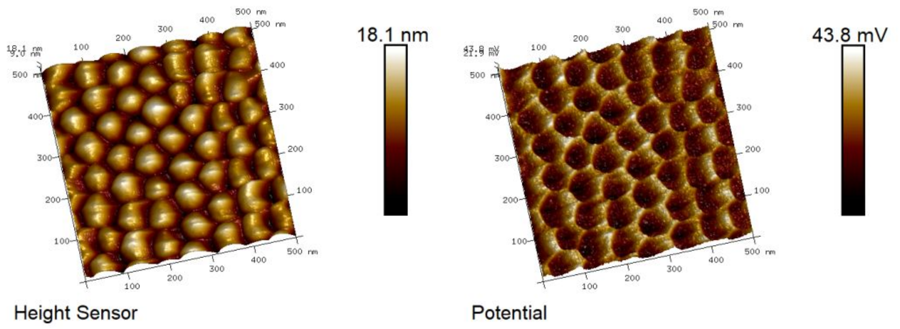

Figure S2. Correspondence of topography and potential of nanoconvex (sample bias).

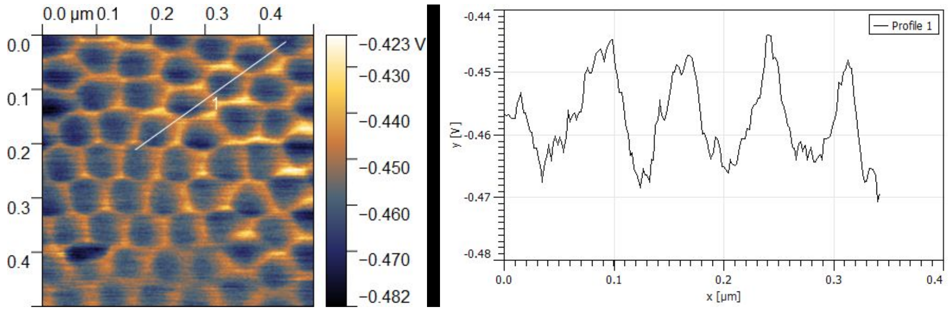

Figure S3. HOPG calibrated potential of nanoconvex (sample bias).

From the KPFM (sample bias) result, the topographical top point A with coordinates

$A(r_0, 0)$, $\varphi(A) = P_A$ and topographical bottom point B with coordinates $B(r_0,$ $arccos3/5)$, $\varphi(B) = P_B$ have,

$$\varphi(A) = -460mV = -0.46V$$

$$\varphi(B) = -440mV = -0.44V$$

Then we have,

$$b_1 = 0.05{r_0}^2$$

$$b_0 = 0.51r_0$$

where

$$\varphi(r, \theta) = \frac{-0.51r_0}{r} + \frac{0.05{r_0}^2}{r^2} cos\, \theta$$

The electric field of nanoconvex feature can be written as follow:

$$E_r = -\frac{\partial \varphi}{\partial r} = \frac{-0.51r_0}{r^2} + \frac{0.1{r_0}^2 cos\, \theta}{r^3}$$

$$E_\theta = -\frac{\partial \varphi}{\partial \theta} = sin\, \theta \frac{0.05{r_0}^2}{r^2}$$

then we have

$$E_A^2 = E_{r_0}^2 + E_0^2 = (\frac{-0.51r_0}{{r_0}^2})^2 + (\frac{0.1}{r_0})^2 + 2\frac{-0.51r_0 0.1}{{r_0}^2 \quad r_0}$$

$$E_B^2 = E_{r_0}^2 + E_{arccos3/5}^2 = (\frac{-0.51r_0}{{r_0}^2})^2 + (\frac{0.06}{r_0})^2 + \frac{0.12 \times -0.51r_0}{r_0^3} + 0.0016$$

$$E_A^2 - E_B^2 = \frac{0.0064}{{r_0}^2} + \frac{0.08 \times -0.51r_0}{{r_0}^3} - 0.0016 < 0$$

Here, the nanoconvex $r_0 = 50\, nm = 5 \times 10^{-8}\, m$,

As one of the basic and important assumption is that the titanium surface is negatively charged, with the boundary conditions of $\varphi(A) < 0$, $\varphi(B) < 0$ then we have,

$$b_0 < -0.05r_0$$

then

$$E_A^2 < E_B^2$$

For surface charge density we have

$$\sigma_A = \varepsilon E_A < \sigma_B = \varepsilon E_B$$

This indicates the electrons distributed at nanoconvex top (A area) has less density than that at $\theta = arccos\,3/5$ area (B), that $\theta = arccos\,3/5$ area can generate more attractive Coulomb's force to charged proteins. However, this equilibrium of electrons distribution is based on without introduce of external charged proteins, electrons are prone to transfer and redistribute while the proteins inducement.

Nanoconcave case:

$$\varphi(r,\theta) = \frac{b_0}{r} + \frac{b_1}{r^2}\cos\theta$$

where, b_0 and b_1 are undetermined coefficients.

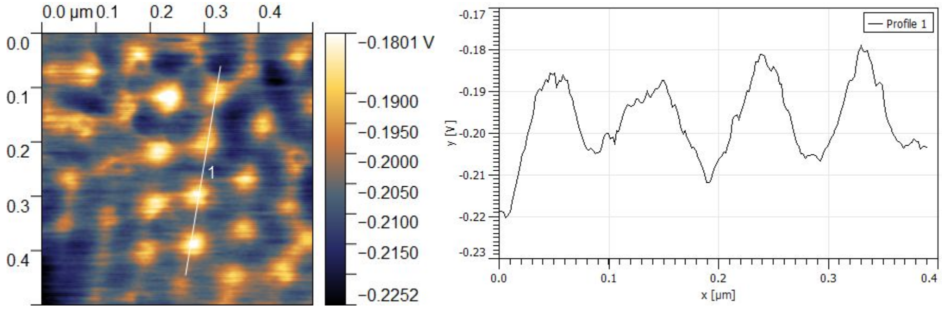

Figure S4. HOPG calibrated potential of nanoconcave (sample bias).

From the KPFM (sample bias) result, the topographical top point A with coordinates $A(r_0, \pi)$, $\varphi(A) = P_A$ and topographical bottom point B with coordinates $B(r_0, \pi - arccos3/5)$, $\varphi(B) = P_B$ have,

$$\varphi(A) - \varphi(B) = 18.92\ mV = 0.019\ V = 0.02V$$

$$\varphi(A) = -185mV = -0.19V$$

$$\varphi(B) = -205mV = -0.21V$$

Then we have,

$$b_1 = -0.05{r_0}^2$$

$$b_0 = -0.24r_0$$

where

$$\varphi(r,\theta) = -\frac{0.24r_0}{r} - \frac{0.05{r_0}^2}{r^2}cos\,\theta$$

The electric filed can be written as follow:

$$E_r = -\frac{\partial \varphi}{\partial r} = -\frac{0.24r_0}{r^2} - \frac{0.1{r_0}^2 cos\,\theta}{r^3}$$

$$E_\theta = -\frac{\partial \varphi}{\partial \theta} = -sin\,\theta\frac{0.05{r_0}^2}{r^2}$$

then we have

$$E_A^2 = E_{r_0}^2 + E_0^2 = (\frac{-0.24r_0}{{r_0}^2})^2 + (\frac{0.1}{r_0})^2 + 2\frac{-0.24r_0 0.1}{{r_0}^2 \quad r_0}$$

$$E_B^2 = E_{r_0}^2 + E_{\pi - arccos3/5}^2 = (\frac{-0.24r_0}{{r_0}^2})^2 + (\frac{0.06}{r_0})^2 + \frac{-0.24r_0 0.12}{{r_0}^2 \quad r_0} + 0.0016$$

$$E_A^2 - E_B^2 < 0$$

This means the electrons distributed at nanoconcave bottom (A area) has less density than that at $\theta = \pi - arccos3/5$ area (B), that $\theta = \pi - arccos3/5$ area can generate more attractive Coulomb's force to charged proteins.

The dynamic distance between nanoconvex/nanoconcave and fibronectin

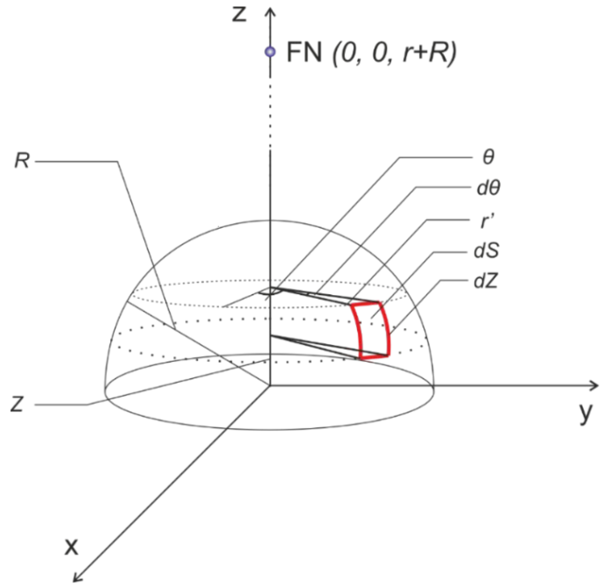

Figure S5. The coordinate illustration of Coulomb's force on vertically vector, FC.

The red box is defined as a 'diminutive area'.

The Coulomb's force between fibronectin and a random area on nanoconvex could be

described as Coulomb's law below;

$$dF = k_e \frac{QQ_{FN}}{d^2}$$

Due to the nanoconvex is symmetric about both x, y axis, the Coulomb's force on horizontal vector can be cancelled out if the fibronectin adsorbed from the centre of nanoconvex. The Coulomb's force on vertically vector, FC can be described as below;

$$dF_{Vertical} = k_e \frac{QQ_{FN}}{d^2} \cdot \frac{r + R - Z}{d}$$

Where,

$k_e = 9 \times 10^9 Nm^2C^{-2}$,

Q_{FN} is the charge of fibronectin, Q_{FN}

Q is the surface charge of a random area on nanoconvex feature,

d is the distance between fibronectin and the random area,

r is the distance between fibronectin and the top surface of nanoconvex feature,

R is the radius of nanoconvex feature,

Z is the vertical coordinate of the lower side of the random area.

In terms of surface charge of the random area (marked with red area in Figure S5), due to the nanoconvex spherical surface can be assembled by numerous random curved regions like the random area, and the surface area of this curved region can be approximated calculated as rectangle, with both sides of dZ and $r' \cdot d\theta$, the charge of random area, Q can be illustrated as below;

$$Q = dS \cdot q_a = r' \cdot d\theta \cdot dZ \cdot q_a$$

Where,

r' is the radius of horizontal round section, see Figure S5,

q_a is the surface charge density of TiO_2 material.

Because of geometric relations, the distance between fibronectin and the random area, d, can be written as below;

$$d = \sqrt{(r'cos\theta)^2 + (r'sin\theta)^2 + (R + r - Z)^2}$$

Thus, the Coulomb's force between FN and nanoconvex can be described as the assembling of infinitesimal calculus (the random area), which can be written as;

$$F_C = \iint dF_{Vertical} = \int_0^R \int_0^{2\pi} dF_{Vertical}$$
